# Mercury increases IL-1β and IL-18 secretion and intensifies coronary arteritis in an animal model of Kawasaki disease

**DOI:** 10.3389/fimmu.2023.1126154

**Published:** 2023-04-14

**Authors:** Martin P. Alphonse, Trang T. Duong, Suzanne Tam, Rae S. M. Yeung

**Affiliations:** ^1^ Cell Biology Research Program, The Hospital for Sick Children Research Institute, Toronto, ON, Canada; ^2^ Department of Immunology, University of Toronto, Toronto, ON, Canada; ^3^ Department of Dermatology, Johns Hopkins University School of Medicine, Baltimore, MD, United States; ^4^ Department of Pediatrics, The Hospital for Sick Children, University of Toronto, Toronto, ON, Canada

**Keywords:** Kawasaki disease (KD), inflammasome, mercury, inflammation, coronary arteritis, NLRP3 inflammasome, calcium signaling, ITPKC/IP3-3KC/IP3KC

## Abstract

Kawasaki disease (KD) is a multisystem vasculitis that predominantly targets the coronary arteries in young children. Epidemiological data suggest both environmental and genetic factors contribute to the susceptibility and severity of the disease. Mercury (Hg) is a known environmental pollutant and a Ca^2+^ signaling modulator. Ca^2+^ signaling regulates the activation of NLRP3 inflammasome. Using the *Lactobacillus casei* cell wall extract (LCWE) induced coronary arteritis mouse model of KD; we studied the effect of mercury on inflammasome activation and its impact on the immunopathogenesis of KD. Mercury enhances the expression of inflammasome activation resulting in caspase-1 mediated secretion of IL-1β and IL-18 cytokines. *In vivo*, the administration of mercury together with disease inducing LCWE exacerbates disease resulting in increased incidence and severity of coronary arteritis compared to LCWE alone. Mercury can act as a novel danger signal modulating Ca^2+^ signaling to increase IL-1β and IL-18 secretion and intensifies coronary arteritis in an animal model of KD.

## Introduction

Kawasaki disease (KD) is a multisystem vasculitis that predominantly targets the coronary arteries in infants and young children (1, 2). It is characterized by prolonged fever and multisystem inflammation with polymorphous skin rash, oral mucosal changes (red, cracked lips, and strawberry tongue), non-purulent conjunctival injection, extremity changes (redness and swelling), and cervical lymphadenopathy. Epidemiological data suggest an environmental trigger that causes disease in genetically predisposed individuals (3, 4). During the acute phase of KD, large amounts of pro-inflammatory cytokines, including inflammasome mediated cytokines IL-1β and IL-18, are produced (5). Genetic studies in KD have associated inositol triphosphate 3-kinase C (ITPKC), an enzyme in the Ca^2+^ signaling pathway, with susceptibility and severity of disease (6). We have shown that ITPKC regulates the secretion of IL-1β and IL-18 in KD (5), suggesting that KD is an inflammasome mediated disease.

Various environmental agents have been associated with outbreaks of KD (7). Environmental triggers include both infectious and non-infectious origins. One such non-infectious environmental pollutant associated with the pathogenesis of KD is mercury (8). Mercury (Hg) is a known Ca^2+^ signaling modulator (9–11). Ca^2+^ signaling regulates the activation of NLRP3 inflammasome. Mercury is a natural component of our environment, making about 0.5 parts per million of the earth’s crust. It is found in three main forms: elemental mercury or quicksilver (Hg°), inorganic mercury (Hg^+^ and Hg^2+^ ), and organic (such as methyl, ethyl, and phenyl) mercury. Inorganic mercury is derived from the oxidization of elemental mercury, while the biomethylation of inorganic mercury compounds produces the organic derivatives by microorganisms in aquatic sediments and soils. The human body can absorb all three forms of mercury. However, the human population is primarily exposed to Hg° and organic mercury, which are converted to inorganic mercury *in vivo* (12).

LCWE-induced coronary arteritis in mice is an animal model for KD. It is similar to children with KD in its time course, susceptibility in the young, vessel pathology, and response to IVIG therapy. Using inorganic mercuric chloride (HgCl2) as a novel danger signal, we studied its role in NLRP3 expression and secretion of IL-1β and IL-18 *in vitro* and *in vivo*. HgCl2 treatment enhances the expression of NLRP3 and secretion of IL-1β and IL-18 cytokines in LCWE-primed bone marrow derived macrophages (BMDM). The HgCl2 mediated secretion of IL-1β and IL-18 is caspase-1 mediated.

Furthermore, we show that extracellular and intracellular Ca^2+^ mobilization is critical for HgCl2 mediated secretion of IL-1β and IL-18. LCWE-injected mice exposed to HgCl2 produce more IL-1β and IL-18 compared to those given HgCl2 or LCWE alone. Additionally, HgCl2 intensifies coronary arteritis *in vivo* with increased incidence and severity of the disease. These results, together with our previous work, show that Ca^2+^ signaling is critical in KD immunopathogenesis and that genetic (ITPKC) and environmental (Hg) factors can directly contribute to the regulation of intracellular calcium mobilization affecting susceptibility to and severity of KD.

## Results

### Mercury is non-toxic to immune cells at low concentrations

**Figure 1:**
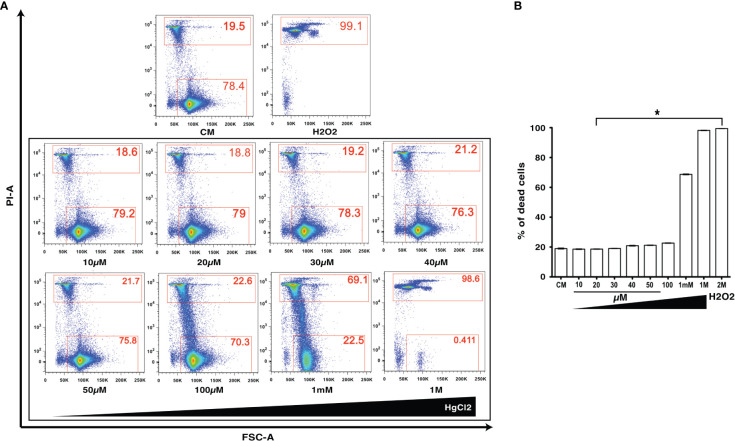
Low concentrations of HgCl2 are non-toxic to immune cells. Splenocytes from C57BL/6 mice were treated with various concentrations of HgCl2 (10μM-1M) for 45 min. Cells were then loaded with Propidium Iodide (PI) and gated for live and dead cells, and assayed by Fluorescence Activated Cell Sorting (FACS). Treatment of cells with H2O2 (2M) was used as a positive control for cell death. CM-Culture Media. **(A)** Representative FACS plots of HgCl2 treated live and dead cells. The X-axis represents Forward Side Scatter Area (FSC-A), and Y- axis represents PI-Area. The data shown are representative of 3 experiments. **(B)** Bar graph showing the % of dead cells (n=3) for various treatments of HgCl2. *p<0.0001.

HgCl2 is a known toxic agent that can cause cell death at high concentrations (10, 11). To determine the cell toxicity of HgCl2 on immune cells, we treated mouse splenocytes with various concentrations (10μM to 1M) of HgCl2. Incorporation of propidium iodine as a marker of cell death was then determined by flow cytometry. Low concentrations of HgCl2 up to 100μM do not cause cell death (**Figures 1A, B**). Non-toxic doses under 20μM were used in this study for *in vitro* experiments.

### Mercury increases IL-1β and IL-18 secretion in LCWE-primed cells

**Figure 2:**
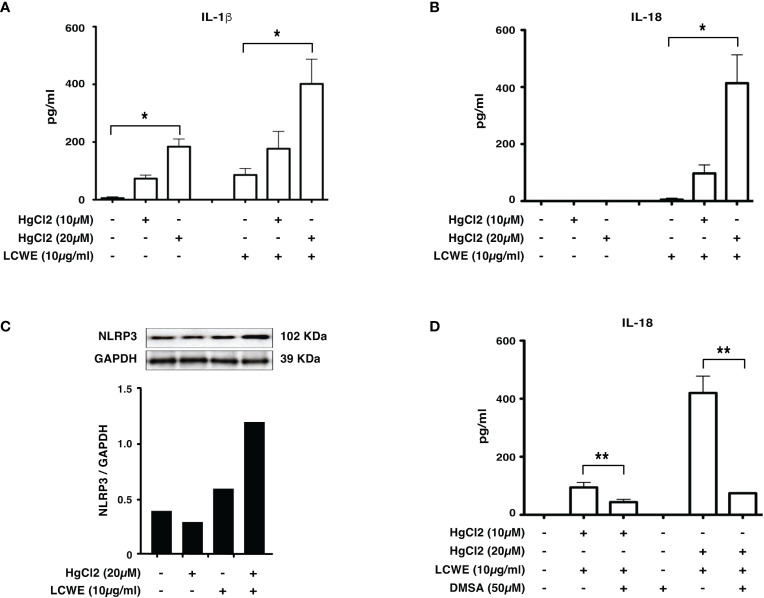
HgCl2 increases NLRP3 expression and increases IL-1β, IL-18 secretion in LCWE-primed immune cells. BMDMs from C57BL/6 mice were treated with LCWE (10mg/ml) for 18 hrs. 30-45 min prior to 18 hrs, cells were treated with/without HgCl2 (10μM or 20μM). Secretion of IL-1β and IL-18 in supernatants was measured by ELISA, and protein expression of NLRP3 in cells was determined by Western blot analysis. **(A)** Levels of IL-1β from supernatants of BMDMs, measured by ELISA (n=3). *p<0.0001. **(B)** Levels of IL-18 from supernatants of BMDMs, measured by ELISA (n=3). *p<0.0001. **(C)** Representative western blot of NLRP3 protein expression from BMDMs. Densitometry was measured, and relative amount of protein normalized to GAPDH are shown in the bar graphs. **(D)** Levels of IL-18 from supernatants of BMDMs, that were treated with/without Hg chelating agent DMSA (50μM) (n=3). **p<0.005.

To determine if mercury could regulate the secretion of IL-1β and IL-18, we treated LCWE-primed BMDMs with HgCl2. HgCl2 increases the secretion of IL-1β cytokine in LCWE-primed BMDMs (**Figure 2A**) compared to LCWE or HgCl2 treatment alone. Similar to previous observations (13), stimulation with LCWE alone does not lead to the secretion of IL-18. However, when LCWE-primed BMDMs were treated with HgCl2, there was the secretion of IL-18 cytokine. IL-18 secretion increases in a dose-dependent fashion with increasing concentrations of HgCl2 (**Figure 2B**). Furthermore, treatment with LCWE but not HgCl2 increases the protein expression of NLRP3 (**Figure 2C**). When LCWE-primed cells were treated with HgCl2, there is increased protein expression of NLRP3 from BMDMs (**Figure 2C**). When HgCl2 was chelated using Dimercaptosuccinic Acid (DMSA) (**Figure 2D**) or Dimercaptopropionic Acid (DMPS) (data not shown), secretion of inflammasome mediated cytokines was significantly reduced. These results suggest that HgCl2 amplifies activation in LCWE-primed immune cells leading to the secretion of inflammasome mediated cytokines IL-1β and IL-18.

### Mercury is a novel danger signal leading to the production of IL-1β and IL-18

**Figure 3:**
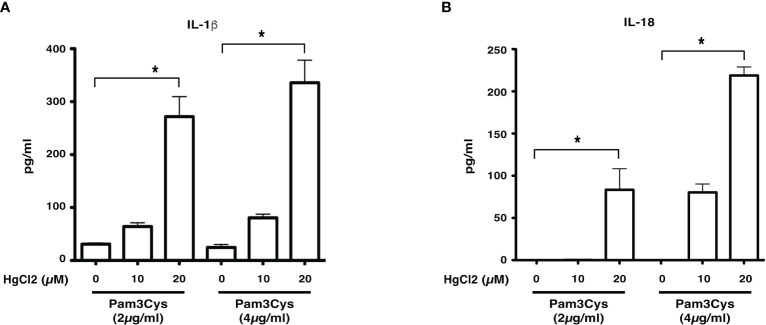
HgCl2 is a novel danger signal mediating the secretion of IL-1β and IL-18. BMDMs from C57BL/6 mice were treated with TLR-2 ligand Pam3Cys (2μg/ml or 4μg/ml) for 18 hrs. 30-45 min prior, cells were treated with/without HgCl2 (20μM). Secretion of IL-1β and IL-18 in supernatants was measured by ELISA. **(A)** Levels of IL-1β from supernatants of BMDMs, were measured by ELISA (n=3). **(B)** Levels of IL-18 from supernatants of BMDMs, measured by ELISA (n=3). *p<0.005.

Previous studies show that LCWE contains a TLR2 ligand (14). To determine if HgCl2 treatment could activate inflammasomes with a known TLR2 ligand, we used Pam3Cys to prime BMDMs. Similar to LCWE, treatment of HgCl2 with Pam3Cys primed cells leads to the secretion of both IL-1β and IL-18 cytokines (**Figures 3A, B**). Taken together, the results suggest that HgCl2 at low concentrations could act as a novel danger signal in providing optimal activation for the secretion of IL-1β and IL-18.

### Mercury treatment increases intracellular Ca^2+^ mobilization

**Figure 4:**
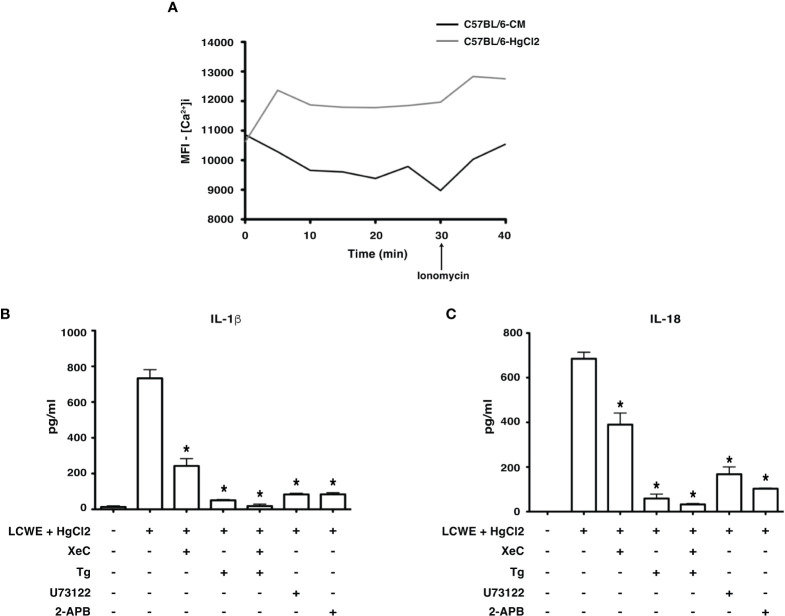
HgCl2 regulates Ca^2+^ mobilization leading to secretion of IL-1β and IL-18. **(A)** [Ca^2+^ ]i levels in treated with/without HgCl2. BMDMs from C57BL/6 were loaded with calcium indicator dye Fluo-4AM and treated with/without HgCl2 (20μM). Live [Ca^2+^ ]i levels were continuously measured using confocal spinning disc microscopy for 40 minutes. Ionomycin (1μM) was added at 30min. The average Mean Fluorescence Intensity (MFI) (Y-axis) from 5 independent fields was measured and plotted against time on the X-axis. The data shown is a representative graph of 2 experiments. BMDMs from C57BL/6 mice were treated with LCWE (10μg/ml) and treated with/without HgCl2 (20μM). Prior to the treatment of HgCl2, cells were treated with/without Ca^2+^ inhibitors XeC (5μM), Tg (100nM), U73122 (10μM), and 2-APB (100μM). **(B)** Levels of IL-1β from supernatants of BMDMs, measured by ELISA (n=3), *p<0.05. **(C)** Levels of IL-18 from supernatants, measured by ELISA (n=3), *p<0.05. Statistical analysis between cultures of LCWE + HgCl2 - stimulated BMDM with/without Ca^2+^ inhibitors is done using the Unpaired t-test.

HgCl2 is a known agent of Ca^2+^ mobilization (15). Studies have shown that Ca^2+^ mobilization is critical for NLRP3 inflammasome activation (5, 16–19). Therefore, to assess whether HgCl2 mediated inflammasome activation is Ca^2+^ dependent, we used extracellular and intracellular Ca^2+^ inhibitors and measured the secretion of IL-1β and IL-18. When live BMDMs from C57BL/6 were assessed for intracellular Ca^2+^ levels using confocal spinning disc microscopy, HgCl2 treated cells acquired more intracellular Ca^2+^ when compared to untreated cells (**Figure 4A**). We next examined whether Ca^2+^ mobilization plays a role in HgCl2 mediated activation of the inflammasome.

### Mercury mediated production of IL-1β and IL-18 is dependent on extracellular Ca^2+^ mobilization

Extracellular Ca^2+^ channels were blocked using Ca^2+^ channel inhibitor 2-APB and PLC pathway inhibitor U73122. Both IL-1β and IL-18 production in cells treated with 2-APB and U73122 was significantly reduced (**Figures 4B, C**), suggesting that extracellular Ca^2+^ mobilization is vital for HgCl2 mediated IL-1β and IL-18 secretion in LCWE-primed BMDM.

### Mercury mediated production of IL-1β and IL-18 is dependent on intracellular Ca^2+^ mobilization

Intracellular Ca^2+^ channels and receptors were blocked using Ca^2+^ channel inhibitor Tg and IP3R-specific inhibitor XeC, respectively. Tg and XeC treatment inhibited the secretion of IL-1β and IL-18 cytokines (**Figure 4C**). The secretion of both IL-1β and IL-18 in cells treated with both extracellular and intracellular Ca^2+^ inhibitors was drastically downregulated (**Figures 4B, C**). These results emphasize the critical role of both extracellular and intracellular Ca^2+^ mobilization in HgCl2 mediated activation of inflammasomes, possibly NLRP3 inflammasome.

### Mercury induced secretion of IL-1β is caspase-1 dependent and NLRP3 mediated

**Figure 5:**
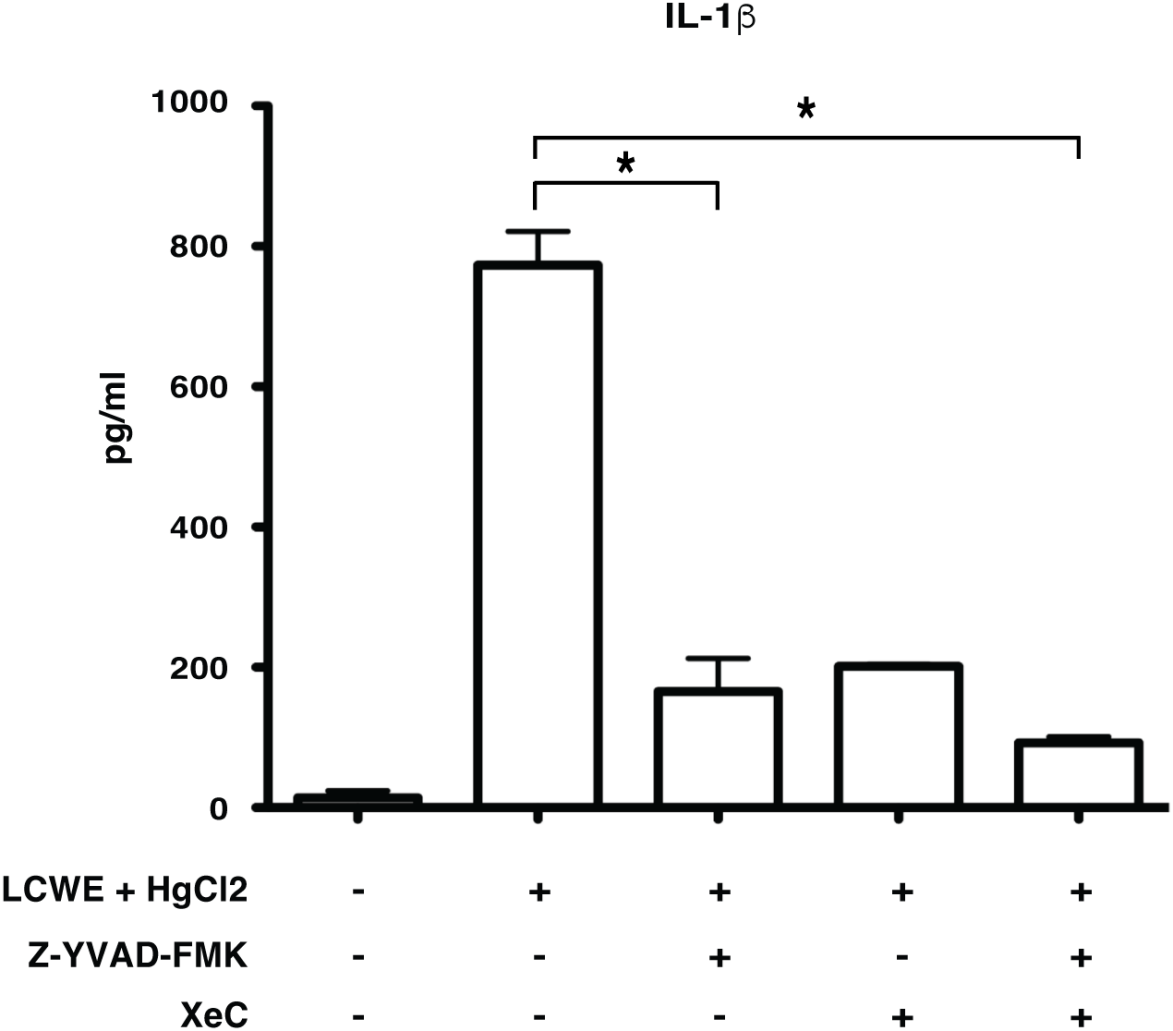
Mercury induced secretion of IL-1β is caspase-1 dependent. BMDMs from C57BL/6 mice were treated with LCWE (10μg/ml) for 18 hrs. 30-45 min prior to 18 hrs, cells were treated with/without HgCl2 (20μM). Prior to the treatment of HgCl2, cells were treated with/without Caspase-1 inhibitor Z-YVAD-FMK (40μM) in combination with/without Ca^2+^ inhibitor XeC (5μM). Levels of IL-1β from supernatants were measured by ELISA (n=3), *p<0.005.

**Figure 6:**
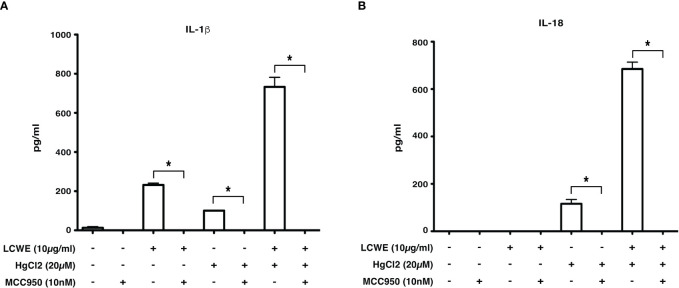
Inhibition of NLRP3 abrogates IL-1β and IL-18 cytokine production. BMDMs from C57BL/6 mice were stimulated with LCWE (10μg/ml) and with/without HgCl2 (20μM). In some cultures, the NLRP3 inhibitor MCC950 was added at 10nM 20-30 minutes prior to the addition of HgCl2. Secretion of IL-1β **(A)** and IL-18 **(B)** in supernatants was measured by ELISA (n=3, *p<0.0001).

Inflammasome mediated secretion of IL-1β and IL-18 cytokines are caspase-1 mediated (20). Therefore, to determine if HgCl2 induced secretion of IL-1β is caspase-1 dependent, Z-YVAD- FMK, a known caspase-1 specific inhibitor, was used. LCWE-primed and HgCl2-activated BMDMs treated with the inhibitor produced significantly less IL-1β cytokine than untreated cells (**Figure 5**). As HgCl2 mediated activation is Ca^2+^ dependent, we treated cells with XeC, a specific inhibitor for IP3R and Z-YVAD-FMK. There was further inhibition of secreted IL-1β cytokine (**Figure 5**) compared to Z-YVAD-FMK or XeC treatment alone. These results show that HgCl2 induced secretion of IL-1β is caspase-1 mediated and is IP3R- dependent. Furthermore, when NLRP3 inflammasome activation is inhibited with a specific NLPR3 inhibitor MCC950 in LCWE with or without HgCl2-treated BMDMs, there is a complete abrogation of IL-1β and IL-18 cytokines secretion (**Figures 6A, B**).

### Mercury exacerbates coronary artery disease in an animal model of KD

**Figure 7:**
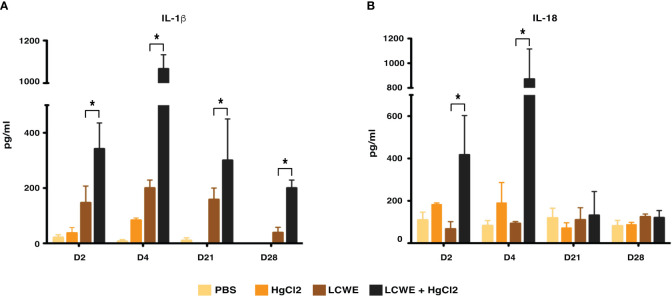
HgCl2 exacerbates secretion of IL-1β and IL-18 in LCWE treated mice *in vivo*. C57BL/6 mice were injected with PBS (1ml, i.p), LCWE (1mg i.p), HgCl2 (1.25 mg/Kg body weight, s.c) or LCWE + HgCl2. Serum was collected on days 2, 4, 21, and 28. Levels of IL-1β **(A)** and IL-18 **(B)** from serum were measured by ELISA (n=5 in each group, *p<0.005).

**Figure 8:**
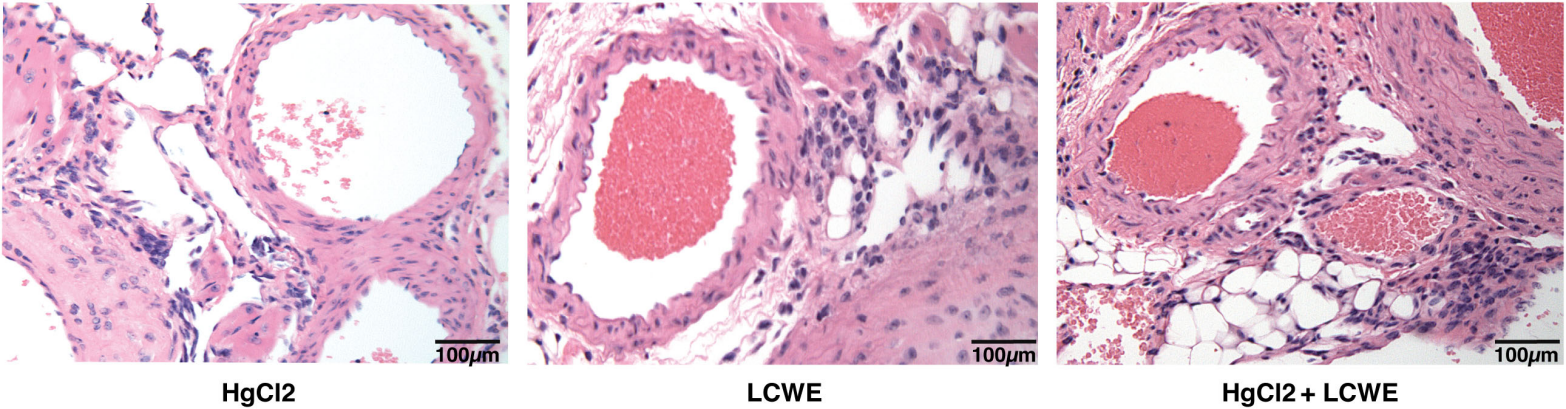
Mercury exacerbates coronary artery disease in LCWE-injected murine model of KD. C57BL/6 mice were injected either with HgCl2 alone (s.c., 1.25mg/Kg body weight), LCWE alone (i.p., 1 mg/mouse) or with both LCWE and HgCl2. Heart sections were prepared 28 days later and stained with haematoxylin and eosin to evaluate the presence of coronary artery inflammation. LCWE-injected and treated with HgCl2 mice exhibited more intense and concentrated cellular infiltrates in comparison to mice injected only with either HgCl2 or LCWE. (200x magnification).

To determine the effect of HgCl2 on the LCWE induced coronary arteritis disease model of KD, we administered HgCl2 to LCWE-injected mice. A single dose of HgCl2 was administered subcutaneously concurrent with LCWE. We assayed circulating IL-1β and IL-18 levels serially and determined the incidence and severity of coronary arteritis as per histologic grading protocol. In mice given LCWE and HgCl2, there was increased production of both IL-1β and IL-18 in the peripheral blood starting at 48 hrs post injection (**Figures 7A, B**). Treatment with mercury also resulted in more severe coronary arteritis. In comparison to mice injected with LCWE alone, those injected with LCWE and HgCl2 exhibited more severe and concentrated cellular infiltrates in the cardiac tissues. On the other hand, in animals injected with just HgCl2, only minimal cellular infiltrates were observed (**Figure 8**). Furthermore, both disease incidence (n=10/10) and severity (Mean ± SD = 1.95 ± 0.15, p<0.0001) were significantly increased in mice injected with LCWE and HgCl2 when compared to LCWE (n=7/10) (Mean ± SD = 0.65 ± 0.47), or HgCl2 (n=0/10) injected mice alone (n=10 in each group) (**Table 1**).

**Table 1:** Mercury increases the incidence and severity of coronary arteritis in mice.

Coronary Arteritis ^a^					
PBS	HgCl2	LCWE		LCWE+HgCl2	
Incidence	Incidence	Incidence	Severity (Mean ± SD)	Incidence	Severity (Mean ± SD)
0/10	0/10	7/10	0.65 ± 0.47	10/10	1.95 ± 0.15^b^

Incidence of coronary arteritis 28 days after disease induction is presented as a total number of mice with histologic evidence of inflammation over the total number of mice injected. Disease severities were significantly increased in mice injected with LCWE and HgCl2 when compared to LCWE alone.

a Cellular infiltrates are evaluated based on a semi-quantitative scoring system from 0 – 4: 0 = no infiltrate; 1 = minimal cellular infiltrate; 2 = moderate cellular infiltrate; 3 = extensive transmural cellular infiltrate; 4 = extensive cellular infiltrate extending well beyond the coronary artery wall. Inflammation was determined based on the presence of monocytes, lymphocytes, and neutrophils, as morphologically identified.

b p<0.0001.

## Discussion

It is well established that genetic factors are associated with KD (7). Genes associated with Ca^2+^ signaling and its pathway have been associated with the severity and susceptibility of KD (6). Alterations in ITPKC increase intracellular Ca^2+^ levels due to increased IP3s (6). Intracellular Ca^2+^ levels are critical for the activation of NLRP3 inflammasome (16). We have shown that ITPKC regulates NLRP3 expression in KD, suggesting that genetic factors that alter Ca^2+^ mobilization or signaling can be involved in the severity of KD (5). Similar to genetic factors, environmental factors can change Ca^2+^ mobilization. Mercury (Hg) is one such environmental factor (12). Both inorganic (HgCl2) and organic (MeHg) forms of mercury can increase intracellular Ca^2+^ mobilization in murine and Jurkat T-cells at low concentrations (10). Furthermore, mercury is one of the many environmental agents associated with KD (9). The clinical symptoms of KD, which form the basis of clinical diagnosis, resemble the clinical features of mercury hypersensitivity in children called Acrodynia or pink disease (8, 9, 21–24).

**Figure 9:**
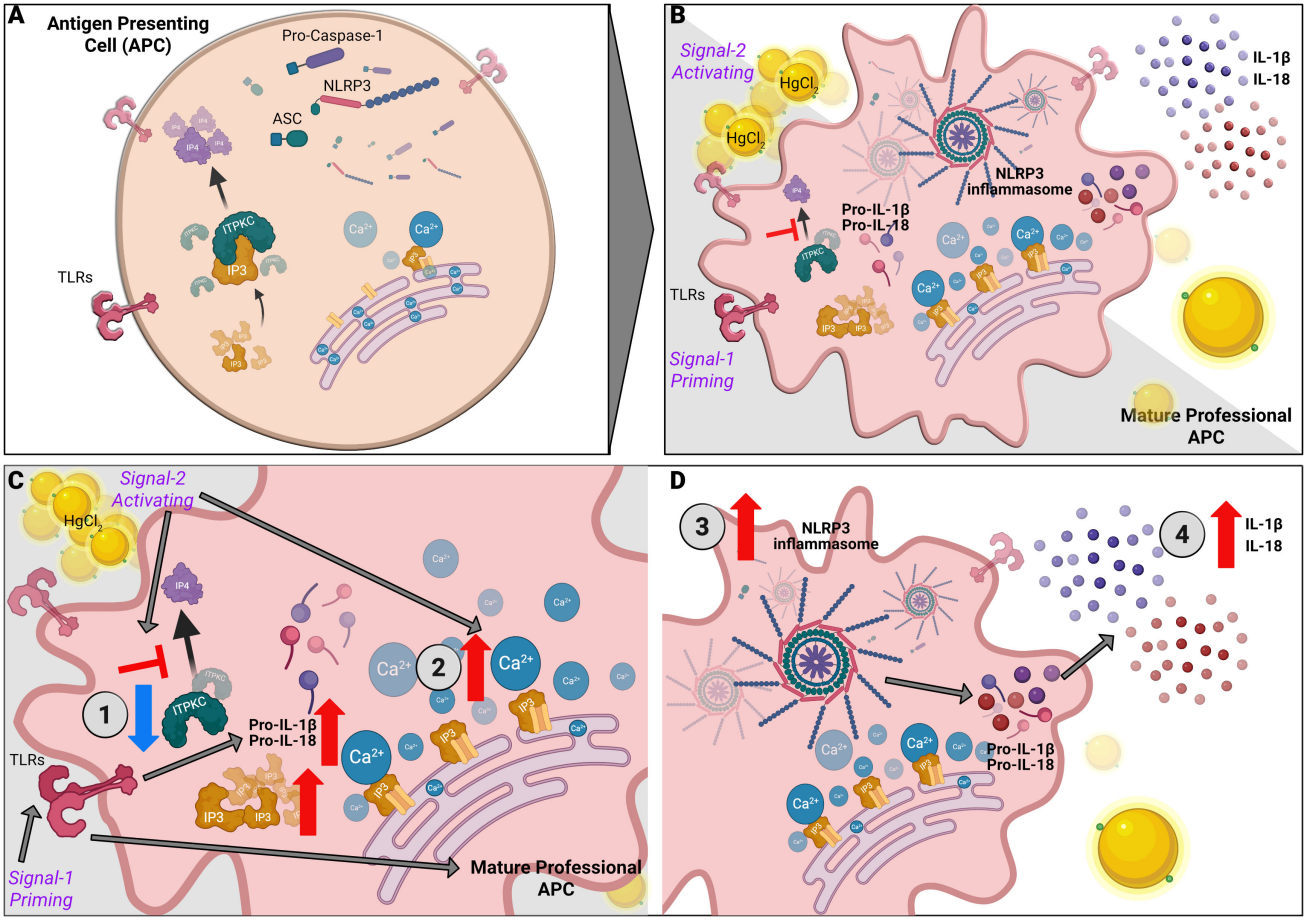
Mercury mediated regulation of Ca^2+^ mediated NLRP3 inflammasome in KD. NLRP3 inflammasome activation is a two-step process with priming and an activation signal. **(A)** Unstimulated APCs. **(B)** Signal one triggers a pattern recognition receptor on APCs such as Toll-like receptors (TLRs) to increase pro-IL-1β and proIL-18 via the NF-κB pathway. Signal two mediates the assembly and activation of the inflammasome, a large molecular platform composed of an NLR protein (such as NLRP3), the adaptor ASC and pro-inflammatory caspases (such as caspase 1). Activation of caspase 1 cleaves pro-IL-1β and pro-IL-18 into their respective active cytokines. The following steps illustrate the contribution of HgCl2 and ITPKC to the disease model: **(C)** 1. ITPKC regulates the phosphorylation of IP3 to IP4. Decreased ITPKC (knockout mice or humans with CC genotype) results in increased IP3, which binds to IP3R. 2. Release of intracellular Ca^2+^ from the endoplasmic reticulum (ER). **(D)** 3. Intracellular Ca^2+^ increases NLRP3 expression and inflammasome activation leading to 4. Increased production of IL-1β and IL-18 from their pro-forms. Mercury acts as signal 2, leading to increased intracellular Ca^2+^ and NLRP3 expression and activation, leading to step 4.

Using mercury as a novel danger signal, we studied the effect of mercury on inflammasome activation in the LCWE induced coronary arteritis murine model of KD. Treating BMDMs with mercury alone does not activate the inflammasome. However, when primed with LCWE, mercury exacerbates the expression of NLRP3 and subsequent secretion of IL-1β and IL-18 cytokines (**Figure 2**). Similar to previous observations (13), LCWE treatment alone leads to activation of NLRP3 and leads to the secretion of IL-1β but not IL-18. Mercury serves as a second signal for inflammasome mediated production of pro-inflammatory cytokines IL-1β and IL-18 (**Figures 2**, **3**). Indeed, when mercury along with LCWE were injected *in vivo*, there is increased secretion of IL- 1β and IL-18 (**Figures 7A, B**). Furthermore, we observed an increase in disease severity and coronary arteritis in mice injected with LCWE and HgCl2 compared to mice injected with a single agent (**Figure 8** and **Table 1**). These results suggest that mercury intensifies KD symptoms in the LCWE mouse model. Previous studies show that extracellular and intracellular Ca^2+^ mobilization is critical for activating the NLRP3 inflammasome (16). Importantly, intracellular Ca^2+^ sensing IP3R receptor is crucial in the activation of the NLRP3 inflammasome (17). Therefore, to further dissect the mechanism of inflammasome activation by HgCl2, we used both intracellular and extracellular Ca^2+^ inhibitors. Tg, XeC are for intracellular channels, where XeC is specific for IP3R receptor and U73122, 2-APB for extracellular Ca^2+^ inhibitors. The activation of IL-1β and IL-18 cytokines after stimulation with LCWE and HgCl2 was dependent on both extracellular and intracellular Ca^2+^, suggesting that HgCl2 mediated activation could be mediated by the NLRP3 inflammasome. Looking back at the past forty years, outbreaks or peak incidences of KD in Japan have been observed to correlate with wind patterns. Peak periods in KD cases coincide with the shift to northwesterly winds originating in central Asia as it crosses Japan and traverses through the North Pacific, suggesting that potential triggering factors for KD may be carried in the wind (25). Industrial pollution of Hg distributed in the atmosphere is measured as geo- anthropogenic emissions, with striking similarities between the geo-anthropogenic emissions of mercury and the prevalence of KD worldwide. As an environmental pollutant, mercury is widely dispersed and can travel long distances in the atmosphere. Interestingly, northwestern winds originating in central Asia would travel through China, the largest producer of mercury in the world (26). With its extensive effects on immune cells and as a known modulator of Ca^2+^ mobilization, mercury may be the environmental trigger contributing to immune activation in KD (**Figure 9**).

The confluence of environmental triggers, both infectious and non-infectious, has taken on added significance during the COVID-19 pandemic, with the emergence of a multisystem inflammatory syndrome in children (MIS-C), a severe phenotype of KD, seen in children as a post-COVID hyperinflammatory syndrome. The key role of inflammasome activation has important implications for therapy, with a growing recognition of the importance of IL-1 blockage in the management of affected children.

## Materials and methods

### Mice

Four weeks old C57BL/6 mice were purchased from The Jackson Laboratory (Bar Harbor, ME). The mice were housed under specific pathogen-free conditions at the University Health Network (Toronto, Canada). All animal studies were performed according to guidelines and procedures approved by Animal Care Committee at both the Hospital for Sick Children Research Institute and the University Health Network (Toronto, Canada).

### Live cell analysis by flow cytometry

Splenocytes (1x10^6^ cells) were treated with various concentrations of HgCl2 (10μM-1M) for 45 minutes. Cells were loaded with propidium iodide (PI) (ThermoFisher) to determine live/dead cells. The cells were acquired using BD^TM^ LSR II Analyzer (BD Biosciences, Mississauga, ON, Canada). Data were analyzed using FlowJo v9 (Tree star, OR, USA) software.

### Bone marrow derived macrophages

BMDMs were obtained by differentiating bone marrow progenitors from the tibia and femur of 8-12 weeks old wild type C57BL/6 mice in RPMI-1640 (VWR, Mississauga, ON, Canada) supplemented with 10% FBS, 1mM Sodium pyruvate, 0.1mM non-essential amino acid, 50µM 2-ME, 2mM L-glutamine and 10mM HEPES, 100 U/ml penicillin and 100µg/ml streptomycin (ThermoFisher, Mississauga, ON, Canada) (complete medium), and 20ng/ml of M-CSF (Sigma Aldrich, MO, USA) for 7 days. The culture medium was replaced with fresh complete medium and M-CSF every 3 days. BMDMs were re-plated in 24 well plates (VWR International) in complete medium at 2.5x10^5^ cells/ml 1 day before experimental assays.

### Activation of IL-1β and IL-18 secretion

BMDMs from C57BL/6 were cultured at 2.5x10^5^ cells/ml in 24 well plates in complete RPMI alone, LCWE (10mg/ml), or Pam3Cys (2 or 4μg/ml) for 18 hours. HgCl2 (10μM-20μM) (Sigma Aldrich) was added during the last 30-45 min of incubation for activation of inflammasomes. Supernatants and cell lysates were collected and assayed for IL1-β and IL-18 by ELISA (eBiosciences, San Diego, CA) and for NLRP3 protein by immunoblotting, respectively. To chelate HgCl2, cells were treated with 50μM meso-2,3-Dimercaptosuccinic acid (DMSA) (Sigma Aldrich).

Intracellular and extracellular Ca^2+^ channels were inhibited with 5μM Xestospongin C (XeC), 100nM Thapsigargin (Tg), 10μM U73122, 100μM 2-Aminoethoxydiphenylborane (2-APB). All Ca^2+^ inhibitors were purchased from Tocris Biosciences, Bristol, UK. For inhibition of caspase-1, irreversible inhibitors Z-YVAD-FMK (40μM) (Abcam, Toronto, ON) and MCC950 (10nM) (Cayman Chemical, Ann Arbor, MI) were used to inhibit NLRP3 inflammasome. Where appropriate, inhibitor agents were added 15-20 minutes prior to the addition of HgCl2.

### *In vivo* studies

Four-week-old C57BL/6 mice were injected with either PBS alone or LCWE (1 mg/mouse) intraperitoneally as per protocol (27). A single injection of 0.1ml HgCl2 (1.25mg/Kg body weight) was administered subcutaneously (28) to animals treated with or without LCWE. Both LCWE and HgCl2 were administered concurrently. Animals were sacrificed on days 2, 4, 21, and 28. Blood was collected, and serum was extracted, aliquoted, and stored at -80oC until use. Heart sections were obtained on day 28, stained with Hematoxylin and Eosin (H&E), and assessed histologically by a blinded reviewer for coronary arteritis. A semi-quantitative score from 0 - 4 was assigned as follows: 0 = no infiltrate; 1 = minimal cellular infiltrate; 2 = moderate cellular infiltrate; 3 = extensive transmural cellular infiltrate; 4 = extensive cellular infiltrate extending well beyond the coronary artery wall. Inflammation was determined based on the presence of monocytes, lymphocytes, and neutrophils, as morphologically identified. LCWE was prepared, and concentration was determined as previously described (27, 29).

### Immunoblots

Proteins from lysates of BMDMs were extracted in Radio-Immunoprecipitation (RIPA) Buffer (Sigma Aldrich) containing Halt^TM^ protease and phosphatase inhibitor cocktail (Fisher Scientific, Napean, ON, Canada). Immunoblots were prepared with Bolt^®^ Bis-Tris Plus Gel (ThermoFisher), and western blot analysis was carried out according to standard protocols, with antibodies specific for rabbit polyclonal raised against human NLRP3 (1:6500, sc-66846, Santa Cruz Biotechnology Inc., Santa Cruz, CA). GAPDH was used as a loading control (AM4300, ThermoFisher). Relative protein levels were normalized to GAPDH as determined by densitometry using Image J software (1.8v, NIH).

### Quantification of secreted cytokines

All ELISAs were carried out as per the manufacturer’s protocol. The following ELISAs were used in this study: Mouse IL-1β and IL-18 in cell culture supernatants or serum were measured using mouse-IL-1β Ready-SET-Go!^®^ and mouse-IL-18 Platinum ELISA kits (ThermoFiasher).

### Calcium analysis by confocal microscopy

BMDMs were plated on 4-chambered glass dishes (Fisher Scientific) at 0.1x10^6^ cells per chamber. Cells were loaded with Fluo-4/AM (ThermoFisher in Ca^2+^ free DMEM media (ThermoFisher) supplemented with 10% FBS, 1mM Sodium pyruvate, 0.1mM non-essential amino acid, 50μM 2-ME, 2mM L-glutamine and 10mM HEPES (complete-DMEM) and maintained in 37oC. Live images of untreated cells were taken at t=0. Cells were then treated with 20μM HgCl2 in Ca^2+^ free DMEM at t=0. Cells were imaged for 40 min with acquisition at 15-sec intervals. Ionomycin (1μM) (Sigma Aldrich) was added to the medium at the end of 30 min, and cells were imaged for the remaining time. Images were acquired using Olympus 1X81^®^ motorized inverted fluorescence microscope with a Hamamatsu C9100-13 black-thinned EM-CCD camera and Yokogawa CSU X1- spinning disc confocal imaging system using the 488-nm laser and emission in the range of 500-600 (Carl Zeiss, Toronto, ON, Canada). Images were analyzed using Volocity software (Perkin Elmer, Woodbridge, ON, Canada). Absolute intensity for cells in 5 independent fields at different time points was obtained, and the average is displayed as the mean fluorescence intensity (MFI) of all cells in the fields.

### Statistical analyses

Paired *t-* test, unpaired *t-* test, or two-way ANOVA were used as appropriate for analysis of both *in vivo and in vitro* experiments using GraphPad Prism v5.0 and v9.0.

## Data availability statement

The raw data supporting the conclusions of this article will be made available by the authors, without undue reservation.

## Ethics statement

The animal study was reviewed and approved by The Hospital for Sick Children Research Institute Animal Care Committee.

## Author contributions

MA and RY designed the study and drafted the manuscript. MA, TD, and ST performed experiments. MA and TD collected and analyzed data. MA, TD, ST, and RY reviewed, revised, and approved the final manuscript. All authors contributed to the article and approved the submitted version.

## References

[1] Kawasaki T Kosaki F Okawa S Shigematsu I Yanagawa H. A new infantile acute febrile mucocutaneous lymph node syndrome (MLNS) prevailing in Japan. Pediatrics (1974) 54(3):271–6. doi: 10.1542/peds.54.3.271 4153258

[2] Yeung RSM. Kawasaki Disease: Update on pathogenesis. Curr Opin Rheumatol (2010) 22(5):551–60. doi: 10.1097/BOR.0b013e32833cf051 20616737

[3] Newburger JW Burns JC. Kawasaki Disease. Vasc Med (1999) 4(3):187–202. doi: 10.1177/1358836X9900400310 10512599

[4] Luca NJC Yeung RSM. Epidemiology and management of Kawasaki disease. Drugs (2012) 72(8):1029–38. doi: 10.2165/11631440-000000000-00000 22621692

[5] Alphonse MP Duong TT Shumitzu C Hoang TL McCrindle BW Franco A. Inositol-triphosphate 3-kinase c mediates inflammasome activation and treatment response in Kawasaki disease. J Immunol (2016) 197(9):3481–9. doi: 10.4049/jimmunol.1600388 27694492

[6] Onouchi Y Gunji T Burns JC Shimizu C Newburger JW Yashiro M. ITPKC functional polymorphism associated with Kawasaki disease susceptibility and formation of coronary artery aneurysms. Nat Genet (2007) 40(1):35–42. doi: 10.1038/ng.2007.59 18084290 PMC2876982

[7] Shulman ST Rowley AH. Kawasaki Disease: Insights into pathogenesis and approaches to treatment. Nat Rev Rheumatol (2015) 11(8):475–82. doi: 10.1038/nrrheum.2015.54 25907703

[8] Orlowski JP Mercer RD. Urine mercury levels in Kawasaki disease. Pediatrics (1980) 66(4):633–6. doi: 10.1542/peds.66.4.633 7432853

[9] Adler R Boxstein D Schaff P Kelly D. Metallic mercury vapor poisoning simulating mucocutaneous lymph node syndrome. J Pedia (1982) 101(6):967–8. doi: 10.1016/S0022-3476(82)80023-1 7143177

[10] Tan X Tang C Castoldi AF Manzo L Costa LG. Effects of inorganic and organic mercury on intracellular calcium levels in rat t lymphocytes. J Toxicol Environ Health (1993) 38(2):159–70. doi: 10.1080/15287399309531709 8433400

[11] Rossi AD Larsson O Manzo L Orrenius S Vahter M Berggren PO. Modifications of Ca^2+^ signaling by inorganic mercury in PC12 cells. FASEB J (1993) 7(15):1507–14. doi: 10.1096/fasebj.7.15.8262335 8262335

[12] Vas J Monestier M. Immunology of mercury. Ann Ny Acad Sci (2007) 1143(1):240–67. doi: 10.1196/annals.1443.022 19076354

[13] Lee Y Schulte DJ Shimada K Chen S Crother TR Chiba N. Interleukin-1β is crucial for the induction of coronary artery inflammation in a mouse model of Kawasaki disease. Circulation (2012) 125(12):1542–50. doi: 10.1161/CIRCULATIONAHA.111.072769 PMC3337219 22361326

[14] Rosenkranz ME Schulte DJ Agle LMA Wong MH Zhang W Ivashkiv L. TLR2 and MyD88 contribute to lactobacillus casei extract-induced focal coronary arteritis in a mouse model of Kawasaki disease. Circulation (2005) 112(19):2966–73. doi: 10.1161/CIRCULATIONAHA.105.537530 16275884

[15] Rajanna B Chetty CS Rajanna S Hall E Fail S Yallapragada PR. Modulation of protein kinase c by heavy metals. Toxicol Lett (1995) 81(2–3):197–203. doi: 10.1016/0378-4274(95)03433-1 8553375

[16] Murakami T Ockinger J Yu J Byles V McColl A Hofer AM. Critical role for calcium mobilization in activation of the NLRP3 inflammasome. Proc Natl Acad Sci USA (2012) 109(28):11282–7. doi: 10.1073/pnas.1117765109 PMC3396518 22733741

[17] Lee GS Subramanian N Kim AI Aksentijevich I Goldbach-Mansky R Sacks DB. The calcium-sensing receptor regulates the NLRP3 inflammasome through Ca^2+^ and cAMP. Nature (2013) 492(7427):123–7. doi: 10.1038/nature11588 PMC4175565 23143333

[18] Rossol M Pierer M Raulien N Quandt D Meusch U Rothe K. Extracellular Ca^2+^ is a danger signal activating the NLRP3 inflammasome through G protein-coupled calcium sensing receptors. Nat Commun (2012) 3:1329. doi: 10.1038/ncomms2339 23271661 PMC3535422

[19] Horng T. Calcium signaling and mitochondrial destabilization in the triggering of the NLRP3 inflammasome. Trends Immunol (2014) 35(6):253–61. doi: 10.1016/j.it.2014.02.007 PMC4041823 24646829

[20] Tschopp J Schroder K. NLRP3 inflammasome activation: The convergence of multiple signalling pathways on ROS production? Nat Rev Immunol (2010) 10(3):210–5. doi: 10.1038/nri2725 20168318

[21] Mutter J Yeter D. Kawasaki’s disease, acrodynia, and mercury. Curr medicinal Chem (2008) 15(28):3000–10. doi: 10.2174/092986708786848712 19075648

[22] Cheek DB. Comment on mucocutaneous lymph node syndrome: Could it be a heavy metal poisoning? Pediatrics (1975) 56(2):335–6. doi: 10.1542/peds.56.2.335b 1161385

[23] Beck C Krafchik B Traubici J Jacobson S. Mercury intoxication: It still exists. Pediatr Dermatol (2004) 21(3):254–9. doi: 10.1111/j.0736-8046.2004.21314.x 15165207

[24] Boyd AS Seger D Vannucci S Langley M Abraham JL King LE. Mercury exposure and cutaneous disease. J Am Acad Dermatol (2000) 43(1 Pt 1):81–90. doi: 10.1067/mjd.2000.106360 10863229

[25] Rodó X Ballester J Cayan D Melish ME Nakamura Y Uehara R. Association of Kawasaki disease with tropospheric wind patterns. Sci Rep (2011) 1:152. doi: 10.1038/srep00152 22355668 PMC3240972

[26] Selin NE. Atmospheric chemistry, modeling, and biogeochemistry of mercury. Mercury Environ: Pattern Process (2012) 159):73–9. doi: 10.1525/california/9780520271630.003.0005

[27] Duong TT Silverman ED Bissessar MV Yeung RSM. Superantigenic activity is responsible for induction of coronary arteritis in mice: An animal model of Kawasaki disease. Int Immunol (2003) 15(1):79–89. doi: 10.1093/intimm/dxg007 12502728

[28] Hu H Möller G Abedi-Valugerdi M. Mechanism of mercury-induced autoimmunity: Both T helper 1- and T helper 2-type responses are involved. Immunology (1999) 96(3):348–57. doi: 10.1046/j.1365-2567.1999.00671.x PMC2326774 10233715

[29] Lehman TJ Walker SM Mahnovski V McCurdy D. Coronary arteritis in mice following the systemic injection of group b lactobacillus casei cell walls in aqueous suspension. Arthritis Rheum (1985) 28(6):652–9. doi: 10.1002/art.1780280609 3924060

